# ADMA in Vascular Disease: More than a Marker?

**DOI:** 10.1371/journal.pmed.0020331

**Published:** 2005-10-04

The endothelium plays a crucial role in the maintenance of vascular tone and structure. Endothelial dysfunction is associated with cardiovascular risk factors, metabolic diseases, and systemic or local inflammation. One proposed mechanism for the development of endothelial dysfunction is the presence of elevated blood levels of asymmetric dimethylarginine (ADMA), an analogue of the amino acid L-arginine. The concentration of ADMA in the plasma of healthy adults varies between 0.4 µM and 1 µM, but it may increase to the range 1.45–4.0 µM in certain diseases.

Elevated ADMA is now widely recognized as a risk marker for vascular disease. Circulating concentrations of ADMA are increased in patients with renal failure, pulmonary hypertension, heart failure, hypercholesterolemia, and a range of other conditions associated with cardiovascular disease. In patients with end-stage renal failure, plasma levels of ADMA have been shown to predict mortality and cardiovascular outcome, and in a cohort of otherwise healthy men, those with the highest levels of ADMA had increased risk of acute coronary events. Increased circulating ADMA in pregnant women predicts increased risk of pre-eclampsia and intrauterine growth retardation.

But is ADMA just a risk marker, or does it have a causal role in the pathophysiology of cardiovascular disease? ADMA inhibits the formation of nitric oxide, a major endothelium-derived vasoactive mediator, and the most potent endogenous vasodilator known. An elevated level of ADMA could, thus, impair vascular function. However, some have argued that the concentration of ADMA found in plasma, even in disease states, is too low to be an effective inhibitor of nitric oxide synthase, and that the usual concentrations of arginine in cells should overcome any inhibitory effects of ADMA on nitric oxide synthase. In order to determine how ADMA might exert effects on endothelial cells and produce pathology, Caroline Smith and colleagues assessed the effects of ADMA on gene expression. [Fig pmed-0020331-g001]

**Figure pmed-0020331-g001:**
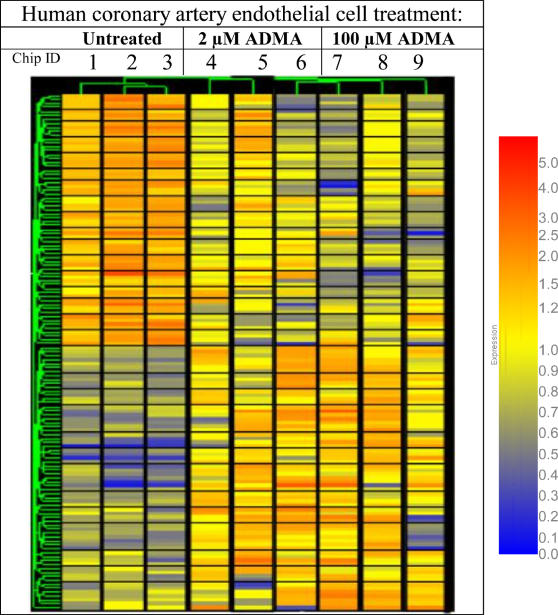
ADMA's effects on endothelial gene expression

The researchers treated human coronary artery endothelial cells with ADMA, and used microarrays to measure the effects on gene expression. They detected substantial changes in gene expression in these cells after 24 hours of exposure to concentrations of ADMA similar to those reported in pathophysiological states. Changes in several genes were confirmed by Northern blotting, quantitative PCR, and in some instances, at the protein level, by Western blotting. To determine whether such changes also occur in vivo, the team examined tissues from mice with elevated ADMA levels. Some of the genes exhibiting consistent changes pointed to pathways known to be associated with cardiovascular risk and pulmonary hypertension.

Smith and colleagues concluded that the concentrations of ADMA found in disease states affect the transcriptional profile of endothelial cells. Moreover, their results suggest novel mechanisms by which ADMA might contribute to or cause disease. Changes in bone morphogenetic protein signaling, and in enzymes involved in arginine methylation, may be particularly relevant to understanding the pathophysiological significance of raised ADMA levels. The effects on bone morphogenetic protein signaling may be important in renal disease and in the link between raised ADMA and pulmonary hypertension. The hope is that in the long-term understanding the mechanisms by which increased ADMA contributes to cardiovascular diseases might suggest new therapeutic strategies.

